# Supplementary material for: Continuously trapped matter-wave interferometry in magic Floquet-Bloch band structures
Source: Nat Commun. 2026 Feb 9;17:2530. doi: 10.1038/s41467-026-69299-y (PMC12996543; doi:10.1038/s41467-026-69299-y)
Supplement: Supplementary file 1 — Supplementary Information [file 41467_2026_69299_MOESM1_ESM.pdf]

Supplementary Information: Continuously trapped matter-wave interferometry in magic Floquet-Bloch band structures

CONTENTS

1. Floquet-Bloch Atom Interferometry Theory 2
   1.1. Bloch Bands in a Static Lattice 2
   1.2. Floquet-Bloch Bands in a Driven Lattice 2
   1.3. Interferometer Phases 3
   1.4. Wave Packets 5
   1.5. Geometric Phase 5
   1.6. Dressed State Picture 6

2. Magic Depth Calculation 7

3. Numerical Calculation 8

4. Calibration 9
   4.1. Lattice Depth 9
   4.2. Force 9
   4.3. Modulation Depth 10

5. Stabilization 11

6. Fringe Contrast Reduction 11

7. Initial Momentum and Pulse Duration Tolerance 12

8. Extending the Loop Size 12

9. Acceleration Sensitivity 13

10. Sources of Systematic Error and Noise 14
    10.1. Lattice Depth 14
    10.2. Modulation Depth 15
    10.3. Acceleration Gradient 16
    10.4. Interaction 17
    10.5. Other sources 17

11. Using Heavier Atoms 18

Supplementary References 19

1. FLOQUET-BLOCH ATOM INTERFEROMETRY THEORY

In this section, we derive the phase of our Floquet-Bloch atom interferometer from first principles.

1.1. Bloch Bands in a Static Lattice

Consider non-interacting atoms confined in a one-dimensional optical lattice. We assume a periodic boundary condition and a system size L as an integer multiple of the lattice constant, *i.e.*, $L = N\lambda/2$ where N is a large integer and λ is the lattice wavelength. The single-particle Hamiltonian is given by

$$\hat{H}_0 = \frac{\hat{p}^2}{2M} - V_0 \cos^2 (k_{\mathrm{L}}\hat{x}), \tag{S1}$$

where M is the atomic mass; V_0 is the lattice depth; $k_{\mathrm{L}} = 2\pi/\lambda$ is the lattice laser angular wavenumber; $\hat{x}$ and $\hat{p}$ are position and momentum operators, respectively. Leveraging the discrete translational symmetry

$$\hat{H}_0 (\hat{x} + \lambda/2) = \hat{H}_0 (\hat{x}), \tag{S2}$$

Bloch's theorem gives rise to the eigenstates $|\varphi_{n,q}\rangle$, where $n = 0, 1, 2...$ denote Bloch band indices (equivalent to $S, P, D...$); $q = 2\pi\hbar j/L$ is the quasimomentum; $j = -N/2 + 1, ..., 0, ..., N/2$; and $\hbar$ is the reduced Planck constant. In position space, the eigenstates satisfy

$$\begin{aligned} \varphi_{n,q}(x) &= \langle x|\varphi_{n,q}\rangle = \frac{1}{\sqrt{N}} u_{n,q}(x) \mathrm{e}^{\mathrm{i}qx/\hbar}, \\ u_{n,q}(x) &= u_{n,q}(x + \lambda/2). \end{aligned} \tag{S3}$$

Substituting this ansatz into the time-independent Schrödinger equation results in an eigenvalue problem for the Bloch function $u_{n,q}(x)$:

$$\left[\frac{(-\mathrm{i}\hbar\partial_x + q)^2}{2M} - V_0 \cos^2 (k_{\mathrm{L}}x)\right] u_{n,q}(x) = E_{n,q} u_{n,q}(x). \tag{S4}$$

This eigenvalue problem can be solved numerically to extract both $u_{n,q}(x)$ and the band energy $E_{n,q}$ (Supplementary Fig. 1a). Notice that if we impose the normalization of $u_{n,q}(x)$ as

$$\int_{-\lambda/4}^{\lambda/4} \mathrm{d}x\, u^*_{n,q}(x) u_{n',q}(x) = \delta_{n,n'}, \tag{S5}$$

the Bloch states are orthonormal:

$$\langle \varphi_{n,q}|\varphi_{n',q'}\rangle = \int_{-L/2}^{L/2} \mathrm{d}x\, \varphi^*_{n,q}(x)\varphi_{n',q'}(x) = \delta_{n,n'}\delta_{q,q'}. \tag{S6}$$

In the following discussion, we take q as a continuous variable, which only makes sense in the $L \to \infty$ limit.

1.2. Floquet-Bloch Bands in a Driven Lattice

Next, we consider a continuous and sinusoidal amplitude-modulation of the lattice,

$$\hat{H}_{\mathrm{Mod}}(t) = \frac{\hat{p}^2}{2M} - (V_0 + \delta V \cos \omega t) \cos^2 (k_{\mathrm{L}}\hat{x}), \tag{S7}$$

where δV and ω are the modulation depth and frequency, respectively. This Hamiltonian exhibits a two-fold (spatial and temporal) translational symmetry that gives rise to the Floquet-Bloch states $|\tilde{\varphi}_{l,q}(t)\rangle$ with wavefunctions given by

$$\tilde{\varphi}_{l,q}(x,t) = \langle x|\tilde{\varphi}_{l,q}(t)\rangle = \exp\left[\frac{\mathrm{i}}{\hbar}(qx - \tilde{E}_{l,q}t)\right] \tilde{u}_{l,q}(x,t), \tag{S8}$$

where l is the Floquet-Bloch band index, $\tilde{E}_{l,q}$ is the quasienergy, and $\tilde{u}_{l,q}(x,t)$ is the Floquet-Bloch function that satisfies

$$\tilde{u}_{l,q}(x,t) = \tilde{u}_{l,q}(x+\lambda/2,t) = \tilde{u}_{l,q}(x,t+2\pi/\omega). \tag{S9}$$

Both $\tilde{E}_{l,q}$ and $\tilde{u}_{l,q}(x,t)$ can be obtained from numerically diagonalizing the Floquet Hamiltonian $\hat{H}_{\mathrm{F}}$, defined as

$$\exp\left(-\frac{2\pi \mathrm{i} \hat{H}_{\mathrm{F}}}{\hbar\omega}\right) = \mathcal{T}\exp\left\{-\frac{\mathrm{i}}{\hbar}\int_0^{2\pi/\omega} \mathrm{d}t\, \hat{H}_{\mathrm{Mod}}(t)\right\}, \tag{S10}$$

where $\mathcal{T}$ is the time-ordering operator.

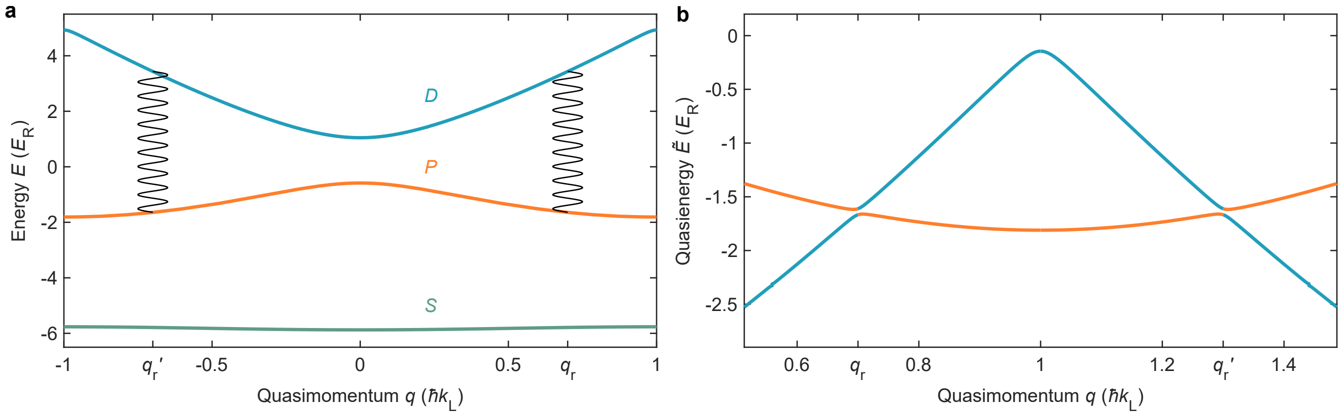

Supplementary Fig. 1. **Band structure**. (**a**) The three lowest bands of a static optical lattice, labeled S (green), P (orange) and D (blue). Amplitude modulation couples the latter two bands at the points of resonance $q_{\mathrm{r}}, q'_{\mathrm{r}}$ found by Eq. (S11), indicated by the connecting "drive photons." (**b**) Floquet-Bloch bands resulting from the amplitude modulation in (a) hybridizing the P and D bands. Orange and blue indicate the dominant character of the band relative to the original static bands.

It is shown in Supplementary Fig. 1b that a resonant modulation opens up gaps between two Floquet-Bloch bands. The modulation frequency ω determines the coupled Bloch band indices (n, n') and the resonant quasimomentum q_{r}, based on the resonance condition

$$E_{n,q_{\mathrm{r}}} - E_{n',q_{\mathrm{r}}} = \hbar\omega. \tag{S11}$$

Meanwhile, the modulation depth δV and the off-diagonal $(n \neq n')$ coupling matrix element govern the size of the gap $\Delta_{q_{\mathrm{r}}}$ at the avoided crossings:

$$\Delta_{q_{\mathrm{r}}} \approx \delta V \left| \langle \varphi_{n,q_{\mathrm{r}}} | \cos^2(k_{\mathrm{L}}\hat{x}) | \varphi_{n',q_{\mathrm{r}}} \rangle \right|. \tag{S12}$$

1.3. Interferometer Phases

When a uniform force $\mathcal{F}$ is applied along the lattice direction, the Hamiltonian reads

$$\hat{H}(t) = \frac{\hat{p}^2}{2M} - (V_0 + \delta V\cos\omega t)\cos^2(k_{\mathrm{L}}\hat{x}) - \mathcal{F}\hat{x}. \tag{S13}$$

Applying a gauge transformation $\hat{U} = \exp(-\mathrm{i}\mathcal{F}t\hat{x}/\hbar)$, we recover the spatial periodicity,

$$\begin{aligned}\hat{H} \to \hat{H}' &= \hat{U}\hat{H}\hat{U}^\dagger + \mathrm{i}\hbar\left(\partial_t \hat{U}\right)\hat{U}^\dagger \\ &= \frac{(\hat{p}+\mathcal{F}t)^2}{2M} - (V_0 + \delta V\cos\omega t)\cos^2(k_{\mathrm{L}}\hat{x}).\end{aligned} \tag{S14}$$

Henceforth, we omit the prime on $\hat{H}'$ and assume $\hat{U}$ is always applied.

We define $\mathcal{Q} = \mathcal{F}t$ as the slowly varying parameter in the Hamiltonian (S14) that facilitates the instantaneous-Floquet-state (IFS) formalism [1]. Since in our experiments the Bloch frequency $\omega_{\mathrm{B}} = \mathcal{F}\lambda/2\hbar \ll \omega$, this is a valid separation of time scales. A set of IFSs $|\tilde{\zeta}_{l,q}(t)\rangle_{\mathcal{Q}}$ can be given by diagonalizing the Hamiltonian (S14) while fixing $\mathcal{Q}$:

$$|\tilde{\zeta}_{l,q}(t)\rangle_{\mathcal{Q}} = |\tilde{\varphi}_{l,q+\mathcal{Q}}(t)\rangle \,. \tag{S15}$$

Intuitively, one would imagine that the quantum state can adiabatically follow the IFS when the control parameter $\mathcal{Q}$ varies slowly. However, an exact Floquet adiabatic limit is likely absent due to the dense quasienergy spectrum and infinitely many avoided-crossings from multi-Floquet-photon resonances [2]. We adopt a coarse-graining argument [3], which states that an infinite number of avoided-crossings below a certain scale can be ignored because of the finite experimental time scale. Away from the sizable avoided-crossings, the quantum state $|\psi(t)\rangle$ still adiabatically follows the IFS in an approximate sense,

$$|\psi(t)\rangle \approx \mathrm{e}^{-\mathrm{i}\phi_{\mathrm{Dyn},l}(t)}\, |\tilde{\zeta}_{l,q_0}(t)\rangle_{\mathcal{Q}} = \mathrm{e}^{-\mathrm{i}\phi_{\mathrm{Dyn},l}(t)}\, |\tilde{\varphi}_{l,q_0+\mathcal{Q}}(t)\rangle \,, \text{ if } \, |\psi(0)\rangle = |\tilde{\varphi}_{l,q_0}(0)\rangle \,, \tag{S16}$$

where

$$\phi_{\mathrm{Dyn},l}(t) = \frac{1}{\hbar\mathcal{F}} \int_{q_0}^{q_0+\mathcal{F}t} \mathrm{d}q \, \tilde{E}_{l,q} \tag{S17}$$

is the dynamical phase, and q_0 is the initial quasimomentum. Combined with the periodicity of quasimomentum, Eq. (S16) confirms the presence of Bloch oscillations in Floquet-Bloch bands. To harness the force sensitivity of $\phi_{\mathrm{Dyn},l}(t)$, we introduce interference between two Floquet-Bloch bands via Landau-Zener tunneling [4–6] at avoided-crossings. This occurs when the Bloch frequency ω_{B} is comparable to the gap size $\Delta_{q_{\mathrm{r}}}/\hbar$. As illustrated in Fig. 1c in the main text, when a Floquet-Bloch atom in the upper band passes the avoided crossing at $q = q_{\mathrm{r}}$, it splits into a superposition of two Floquet-Bloch waves, and the ratio can be adjusted by tuning δV, and thus the gap size, to realize a 50-50 beam splitter. The two waves accumulate distinct dynamical phases before recombining at the second avoided crossing at $q = q'_{\mathrm{r}}$. This setup forms a Landau–Zener–Stückelberg–Majorana interferometer [7, 8], whose output is measured as the final population imbalance between the Floquet-Bloch bands given by the adiabatic-impulse approximation [1]

$$\mathcal{I} = p_{\mathrm{L}} - p_{\mathrm{U}} \approx 4\mathcal{P}(1-\mathcal{P})\cos\phi_{\mathrm{Int}}, \tag{S18}$$

where L(U) denotes the lower (upper) Floquet-Bloch band and $p_{\mathrm{L(U)}}$ represents the band population;

$$\mathcal{P} = \mathrm{e}^{-2\pi\delta} \tag{S19}$$

is the Landau-Zener transition probability;

$$\delta = \frac{\Delta_{q_{\mathrm{r}}}^2}{4\hbar v} \tag{S20}$$

is the adiabaticity parameter;

$$v = \mathcal{F}\sqrt{2\Delta_{q_{\mathrm{r}}}\left|\frac{\partial^2 \tilde{E}_{\mathrm{L},q_{\mathrm{r}}}}{\partial q^2}\right|} \tag{S21}$$

is the Landau-Zener sweep velocity;

$$\phi_{\mathrm{Int}} = \phi_{\mathrm{Dyn}} + 2\phi_{\mathrm{Sto}} \tag{S22}$$

is the interferometer phase;

$$\phi_{\mathrm{Sto}} = -\frac{\pi}{4} + \delta(\ln\delta - 1) + \arg\Gamma(1 - \mathrm{i}\delta) \tag{S23}$$

is the Stokes phase [7, 8], and

$$\phi_{\mathrm{Dyn}} = \frac{1}{\hbar\mathcal{F}} \int_{q_{\mathrm{r}}}^{q'_{\mathrm{r}}} \mathrm{d}q \left(\tilde{E}_{\mathrm{U},q} - \tilde{E}_{\mathrm{L},q}\right) \tag{S24}$$

is the (differential) dynamical phase which is *inversely* proportional to the applied force. Since the Stokes phase ϕ_{Sto} only weakly depends on $\mathcal{F}$ (Supplementary Fig. 2), the force sensitivity of this interferometer is dominated by the dynamical phase ϕ_{Dyn}, which scales with the energy-momentum area enclosed by the interferometer loop.

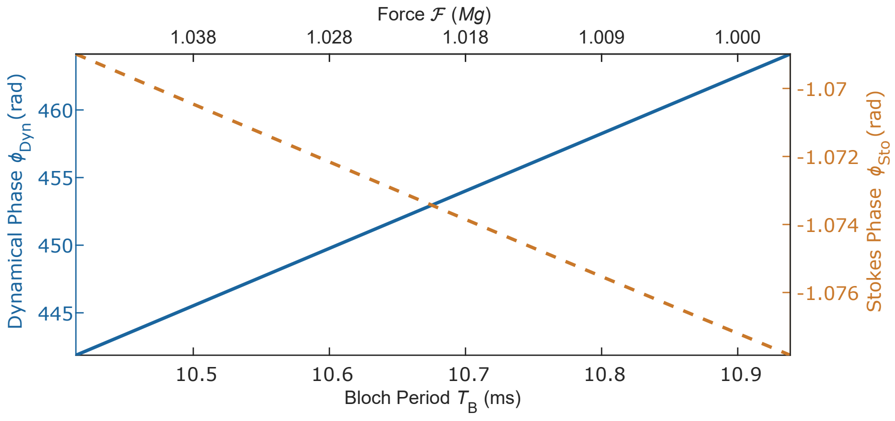

Supplementary Fig. 2. **Interferometer Phases**. The dynamical (blue, solid) and Stokes (brown, dashed) phases as a function of Bloch period. The lattice and modulation parameters are the same as in Fig. 1 in the main text. Note the very different y axes for the different phases; the change in the Stokes phase is generally negligible.

1.4. Wave Packets

The above analysis only applies to a Floquet-Bloch wave with a single quasimomentum component, which is spatially delocalized. Realistically, the quantum state of the atomic cloud in experiments is a localized wave packet, which justifies the following initial condition as a superposition of quasimomentum states,

$$|\psi(0)\rangle = \int_{-\hbar k_{\mathrm{L}}}^{\hbar k_{\mathrm{L}}} \mathrm{d}q_0\, \mathscr{P}(q_0)\, |\tilde{\varphi}_{l,q_0}(0)\rangle\,, \tag{S25}$$

where $\mathscr{P}(q_0)$ is the quasimomentum distribution function. If the quasimomentum distribution is narrow enough, all the quasimomentum components can traverse and complete the interferometric loop, thanks to the robustness of Landau-Zener transitions. In such conditions, the interferometer output, measured as the P and D band population imbalance, is not different from the output with a single quasimomentum component.

We assume a coherent sample for this experiment and discussion, but we believe this model should hold for a thermal ensemble as well if the temperature is low enough that the initial momentum distribution is mostly confined to a single band. In the absence of interaction it should be sufficient that the temperature be this cold only along the lattice axis, which could be achieved with a velocity selective filtering process, as long as the transverse confinement is wide and deep enough that all atoms see about the same lattice depth.

1.5. Geometric Phase

Since the atoms follow an adiabatic path along the IFSs, one may be concerned with the appearance of a geometric phase in the calculation of the interferometer phase. In particular, the analysis of a topologically invariant Zak phase in [9] only applies to static lattices and paths across the entire Brillouin zone, while our considerations require a modulated lattice across part of the Brillouin zone. It is shown in [10] that the geometric phase for periodically driven systems in the extended Hilbert space takes the form

$$\gamma_l(C) = \frac{1}{T}\int_C \mathrm{d}q \int_0^T \mathrm{d}t\, \langle \tilde{u}_{l,q}(t)|\mathrm{i}\partial_q \tilde{u}_{l,q}(t)\rangle, \tag{S26}$$

where $T = 2\pi/\omega$ is the period of the drive; the states $\langle x|\tilde{u}_{l,q}(t)\rangle = \tilde{u}_{l,q}(x,t)$; and C is the path taken through the parameter space, which for our interferometric loop consists of the portion of the Brillouin zone bounded by $q \in [q_{\mathrm{r}}, q_{\mathrm{r}}']$. Then, the differential geometric phase for our two-band interferometer is given by:

$$\gamma_{\mathrm{U}} - \gamma_{\mathrm{L}} = \int_{q_{\mathrm{r}}}^{q_{\mathrm{r}}'} \mathrm{d}q \left[\tilde{X}_{\mathrm{UU}}(q) - \tilde{X}_{\mathrm{LL}}(q)\right], \tag{S27}$$

where the modified Berry connection is given by (S26):

$$\tilde{X}_{ll}(q) = \frac{1}{T}\int_0^T \mathrm{d}t \int_{-\lambda/4}^{\lambda/4} \mathrm{d}x\, \tilde{u}_{l,q}^*(x,t)\left[\mathrm{i}\frac{\partial}{\partial q}\tilde{u}_{l,q}(x,t)\right]. \tag{S28}$$

Numerical calculations of the Zak phase (evaluated along paths spanning the entire Brillouin zone) for various lattice and modulation parameters reveal that the Floquet-Bloch band is topologically trivial ($\gamma = 2\pi\mathcal{N}$ with $\mathcal{N} \in \mathbb{Z}$), so under a time-independent gauge transformation $\tilde{u}_{l,q}(x,t) \to \tilde{u}'_{l,q}(x,t) = e^{\mathrm{i}\tilde{\phi}_l(q)}\tilde{u}_{l,q}(x,t)$, the connection transforms as

$$\tilde{X}_{ll} \longrightarrow \tilde{X}'_{ll} = \tilde{X}_{ll} - \frac{1}{T}\int_0^T \mathrm{d}t\, \frac{\partial\tilde{\phi}_l}{\partial q} = \tilde{X}_{ll} - \frac{\partial\tilde{\phi}_l}{\partial q}, \tag{S29}$$

and likewise the differential geometric phase as

$$\gamma_{\mathrm{U}} - \gamma_{\mathrm{L}} = \int_{q_{\mathrm{r}}}^{q'_{\mathrm{r}}} \mathrm{d}q \left[\tilde{X}'_{\mathrm{UU}}(q) - \tilde{X}'_{\mathrm{LL}}(q)\right]. \tag{S30}$$

A judicious choice of $\tilde{\phi}_l$ can thus be used to gauge away the differential geometric phase by solving the following equation for both the upper and lower bands:

$$\gamma_l = \int_{q_{\mathrm{r}}}^{q'_{\mathrm{r}}} \mathrm{d}q\, \tilde{X}'_{ll}(q) = \int_{q_{\mathrm{r}}}^{q'_{\mathrm{r}}} \mathrm{d}q \left(\tilde{X}_{ll} - \frac{\partial\tilde{\phi}_l}{\partial q}\right) = 0. \tag{S31}$$

Substitution of Equation (S28) for $\tilde{X}_{ll}$ yields:

$$\gamma_l = \frac{1}{T}\int_0^T \mathrm{d}t \int_{q_{\mathrm{r}}}^{q'_{\mathrm{r}}} \mathrm{d}q \int_{-\lambda/4}^{\lambda/4} \mathrm{d}x\, \tilde{u}_{l,q}^*(x,t)\left[\mathrm{i}\frac{\partial}{\partial q}\tilde{u}_{l,q}(x,t)\right] - \left[\tilde{\phi}_l\left(q'_{\mathrm{r}}\right) - \tilde{\phi}_l\left(q_{\mathrm{r}}\right)\right] = 0. \tag{S32}$$

A solution for $\tilde{\phi}_l$ is then obtained:

$$\tilde{\phi}_l\left(q'_{\mathrm{r}}\right) - \tilde{\phi}_l\left(q_{\mathrm{r}}\right) = \frac{1}{T}\int_0^T \mathrm{d}t \int_{q_{\mathrm{r}}}^{q'_{\mathrm{r}}} \mathrm{d}q \int_{-\lambda/4}^{\lambda/4} \mathrm{d}x\, \tilde{u}_{l,q}^*(x,t)\left[\mathrm{i}\frac{\partial}{\partial q}\tilde{u}_{l,q}(x,t)\right]. \tag{S33}$$

Therefore, the geometric phase across an arbitrary portion of the Brillouin zone is pure gauge, so it is not physically observable and we can ignore it in our calculations of the interferometer phase.

1.6. Dressed State Picture

In main text Fig. 4, since we vary the modulation amplitude and phase and include additional modulation frequencies, the Hamiltonian loses perfect discrete time-translation symmetry, so that in principle the Floquet formalism is no longer applicable. This is why we use the phrase "dressed energy" rather than quasienergy here. Nevertheless, the hybridized dressed bands generated by a temporally local Floquet picture are of great assistance in predicting the dynamics and sensitivity of different modulation schemes. Alternatively, the dressed state picture provides a simpler framework for understanding the operation of the interferometer in the absence of discrete time-translation symmetry, as we introduce in the following.

Consider the following Hamiltonian where the amplitude modulation has an envelope $\alpha(t)$:

$$\hat{H}(t) = \frac{\hat{p}^2}{2M} - V_0\left[1 + \alpha(t)\cos\omega t\right]\cos^2\left(k_{\mathrm{L}}\hat{x}\right) - \mathcal{F}\hat{x}. \tag{S34}$$

After a gauge transformation $\hat{U} = \exp(-\mathrm{i}\mathcal{F}t\hat{x}/\hbar)$, the Hamiltonian recovers discrete space-translation symmetry,

$$\hat{H}(t) \to \hat{U}\hat{H}\hat{U}^\dagger + \mathrm{i}\hbar\left(\partial_t\hat{U}\right)\hat{U}^\dagger = \frac{(\hat{p} + \mathcal{F}t)^2}{2M} - V_0\left[1 + \alpha(t)\cos\omega t\right]\cos^2\left(k_{\mathrm{L}}\hat{x}\right). \tag{S35}$$

We assume initially the wave function has the form of a Bloch wave under this gauge,

$$\psi(x, t=0) = \langle x|\psi(t=0)\rangle = \mathcal{U}(x, t=0)\mathrm{e}^{\mathrm{i}q_0 x/\hbar}, \tag{S36}$$

where $\mathcal{U}(x, t=0)$ is periodic in space with periodicity of $\lambda/2$ and q_0 is the initial quasimomentum. Because of the discrete space-translation symmetry of (S35), the quasimomentum is conserved and we can write, at a later time t,

$$\psi(x,t) = \langle x|\psi(t)\rangle = \mathcal{U}(x,t)\mathrm{e}^{\mathrm{i}q_0 x/\hbar}, \tag{S37}$$

where $\mathcal{U}(x,t)$ is also periodic in space. Performing a backward gauge transformation $\hat{U}^\dagger = \exp(\mathrm{i}\mathcal{F}t\hat{x}/\hbar)$, we have

$$\psi(x,t) = \langle x|\psi(t)\rangle \to \psi'(x,t) = \langle x|\hat{U}^\dagger\psi(t)\rangle = \mathcal{U}(x,t)\mathrm{e}^{\mathrm{i}(q_0+\mathcal{F}t)x/\hbar}. \tag{S38}$$

Therefore, in the original gauge the time-dependent wavefunction can be expressed as a superposition of Bloch states with the instantaneous quasimomentum $q = q_0 + \mathcal{F}t$ (omitting the prime symbol for simplicity):

$$|\psi(t)\rangle = \sum_{n=0}^{\infty} c_n(t)\,|\varphi_{n,q_0+\mathcal{F}t}\rangle\,. \tag{S39}$$

In the following, we assume the summation can be truncated to include only two Bloch bands, e.g., the P and D bands:

$$|\psi(t)\rangle \approx \sum_{n=1}^{2} c_n(t)\,|\varphi_{n,q_0+\mathcal{F}t}\rangle\,. \tag{S40}$$

This two-band approximation is valid when we consider dynamics near the P-D resonance driven by the modulation, or when the modulation is weak enough such that it barely couples transitions outside of the P-D subspace. Substituting Eq. (S40) into the time-dependent Schrödinger equation with the Hamiltonian (S34), we have

$$\mathrm{i}\hbar\frac{\partial}{\partial t}\begin{pmatrix} c_2 \\ c_1 \end{pmatrix} = \mathbf{H}_{\mathrm{Eff}}\begin{pmatrix} c_2 \\ c_1 \end{pmatrix},\ \mathbf{H}_{\mathrm{Eff}}(t) \approx \begin{pmatrix} E_{2,q_0+\mathcal{F}t} - \hbar\omega & -\hbar\Omega(t)/2 \\ -\hbar\Omega^*(t)/2 & E_{1,q_0+\mathcal{F}t} \end{pmatrix}, \tag{S41}$$

where the Rabi frequency is $\Omega(t) = \alpha(t)V_0\,\langle\varphi_{1,q_0+\mathcal{F}t}|\cos^2(k_{\mathrm{L}}\hat{x})|\varphi_{2,q_0+\mathcal{F}t}\rangle/\hbar$. To derive the effective Hamiltonian $\mathbf{H}_{\mathrm{Eff}}(t)$, we have applied a rotating-frame transformation and used the rotating-wave approximation (RWA), while neglecting the linear potential term $-\mathcal{F}\hat{x}$, which typically contributes only a small perturbation for P and D bands.

Eq. (S41) implies that the system's dynamics can be understood as those of a two-level system with time-dependent parameters. In fact, the theory of Stückelberg interferometers [7, 8] can be directly applied for such systems. The interferometer loop is formed by two Landau-Zener transitions between the dressed states, and the interferometer phase is dominated by the dynamical phase, given as

$$\phi_{\mathrm{Dyn}} = \frac{1}{\hbar}\int_{t_{\mathrm{r}}}^{t'_{\mathrm{r}}} \mathrm{d}t\,[\epsilon_+(t) - \epsilon_-(t)]\,, \tag{S42}$$

where $\epsilon_\pm(t)$ are the dressed energies obtained by diagonalizing the effective Hamiltonian $\mathbf{H}_{\mathrm{Eff}}(t)$ at each moment in time, and $t_{\mathrm{r}}, t'_{\mathrm{r}}$ correspond to solutions of the resonance condition $E_{2,q_0+\mathcal{F}t} - \hbar\omega = E_{1,q_0+\mathcal{F}t}$. This framework provides an alternative method for computing the interferometer phase that does not rely on exact discrete time-translational symmetry. Moreover, this dressed-state approach can be straightforwardly extended to include multi-tone driving, multi-band dynamics, and varying modulation phase.

2. MAGIC DEPTH CALCULATION

Since the Stokes phase (Eq. (S23)) is monotonic in δ and bounded by $[-\pi/2, -\pi/4]$, it varies far less than the dynamical phase for the same range of force and lattice depth, as shown in Supplementary Fig. 2. Thus, while exploring the parameter space of V_0, q_{r}, and ω, we approximate the magic condition as

$$\begin{aligned} 0 &= \frac{\partial\phi_{\mathrm{Int}}}{\partial V_0} \\ &= \frac{\partial\phi_{\mathrm{Dyn}}}{\partial V_0} + 2\frac{\partial\phi_{\mathrm{Sto}}}{\partial V_0} \\ &\approx \frac{\partial\phi_{\mathrm{Dyn}}}{\partial V_0}. \end{aligned} \tag{S43}$$

We focus on interferometer loops that couple the P and D bands at two quasimomenta q_{r}, q_{r}' which are symmetric about the Brillouin zone edge. In such cases, when the loop size Δq is an integer multiple of $2\hbar k_{\mathrm{L}}$, the resonant quasimomenta q_{r}, q_{r}' fall at the Brillouin zone edge or center, where the modulation coupling strength vanishes. As a result, interferometer loops cannot form when $\Delta q \simeq 2\mathcal{N}\hbar k_{\mathrm{L}}$ with $\mathcal{N} \in \mathbb{Z}^+$, leading to the gaps in Supplementary Fig. 8c and 8d. As indicated in the main text, we have to pulse the modulation to avoid unwanted band couplings when the loop size becomes larger, which formally makes the dressed state approximation necessary. To evaluate the magic condition using dressed energies, we adopt the following two-step protocol.

First, since our amplitude modulation is perturbatively weak, static Bloch band energies well approximate the actual Floquet-Bloch band quasienergies. We use numerical integration of these static band energies across the desired quasimomentum range $[q_{\mathrm{r}}, q_{\mathrm{r}}']$ with sampling resolution 10^3 and numerical differentiation with respect to lattice depth V_0 to estimate the magic condition and the modulation frequency/depth required to couple the P and D bands at the desired quasimomenta.

We then confirm and refine this prediction with a more sophisticated calculation using Eq. (S42) at a fixed modulation frequency and modulation depth (rather than fixed resonant quasimomentum), as in Fig. 2a in the main text. Since both Bloch and Floquet-Bloch bands become flat in the limit of infinite lattice depth, the differential dynamical phase between Floquet-Bloch bands vanishes in the same limit for fixed q_{r}, precluding the possibility of a local minimum in ϕ_{Dyn}. We instead allow q_{r} to vary for fixed modulation frequency; consequently, the ϕ_{Dyn} curves terminate at some minimum and/or maximum lattice depth beyond which the bands are no longer resonant with the modulation for any quasimomentum.

Likewise, numerical calculation of ϕ_{Int} as a function of V_0 and Δq allows estimation of the lattice tolerance in the main text Fig. 3b, defined as the deviation from the magic depth V_0^{M} resulting in $\phi_{\mathrm{Int}}^{\mathrm{M}} \pm \pi/4$.

3. NUMERICAL CALCULATION

In this section, we describe numerical methods for obtaining (Floquet-) Bloch energy bands. We decompose the Bloch function (S3) using Fourier modes:

$$u_{n,q}(x) = \sum_{j=-\infty}^{\infty} c_{n,q}^{(j)} \exp\left(2\mathrm{i}jk_{\mathrm{L}}x\right). \tag{S44}$$

Substituting this ansatz into the Schrödinger equation (S4) gives the eigenvalue equation

$$\sum_{j'=-\infty}^{\infty} \left[\left(\frac{(2j\hbar k_{\mathrm{L}} + q)^2}{2M} - \frac{V_0}{2} \right) \delta_{j,j'} - \frac{V_0}{4} \left(\delta_{j-1,j'} + \delta_{j+1,j'} \right) \right] c_{n,q}^{(j')} = E_{n,q} c_{n,q}^{(j)}, \tag{S45}$$

which can be solved numerically if we truncate the Fourier components up to appropriate limits $\pm j_{\mathrm{Max}}$. Using a similar approach for the Floquet-Bloch function (S8)

$$\tilde{u}_{l,q}(x,t) = \sum_{j,k=-\infty}^{\infty} c_{l,q}^{(j,k)} \exp(\mathrm{i}(2jk_{\mathrm{L}}x - k\omega t)), \tag{S46}$$

results in the eigenvalue equation:

$$\begin{aligned} \tilde{E}_{l,q} c_{l,q}^{(j,k)} = \sum_{j',k'=-\infty}^{\infty} c_{l,q}^{(j',k')} \Bigg\{ & \left(\frac{(2j\hbar k_{\mathrm{L}} + q)^2}{2M} - \frac{V_0}{2} - k\hbar\omega \right) \delta_{j,j'} \delta_{k,k'} \\ & - \frac{V_0}{4} \left(\delta_{j-1,j'} \delta_{k,k'} + \delta_{j+1,j'} \delta_{k,k'} \right) \\ & - \frac{\delta V}{4} \left(\delta_{j,j'} \delta_{k+1,k'} + \delta_{j,j'} \delta_{k-1,k'} \right) \\ & - \frac{\delta V}{8} \left(\delta_{j-1,j'} \delta_{k+1,k'} + \delta_{j+1,j'} \delta_{k+1,k'} \right) \\ & - \frac{\delta V}{8} \left(\delta_{j-1,j'} \delta_{k-1,k'} + \delta_{j+1,j'} \delta_{k-1,k'} \right) \Bigg\}. \end{aligned} \tag{S47}$$

In practice, $c_{l,q}^{(j,k)}$ and the j, j', k, k' indices can be flattened to produce a vector eigenvalue equation where the eigenvalues are the quasienergies. Again, this eigenvalue problem can be solved if we truncate the Fourier components in space and time up to appropriate limits $\pm j_{\mathrm{Max}}$ and $\pm k_{\mathrm{Max}}$. This method is used along with diagonalizing the Floquet Hamiltonian in (S10), and both approaches produce the same numerical results.

4. CALIBRATION

4.1. Lattice Depth

To calibrate the depth of our optical lattice, we perform amplitude modulation spectroscopy on the $S \to D$ band transition at zero quasimomentum. To do this, we adiabatically load the BEC from the optical dipole trap into the optical lattice (Supplementary Fig. 3a) and then perform amplitude modulation with a 1 kHz frequency sweep over 2 ms. If the band energy difference between the S and D bands at $q = 0$ falls into the sweep range, atoms will be excited into the D band; after band mapping, the higher-momentum D band atoms are separated from the S band (zero-momentum) atoms to count their relative populations. Scanning the center frequency of this sweep (Supplementary Fig. 3b) produces a resonance peak, from which we can extract the band energy difference by fitting. The statistical error of characterizing the resonant frequency is typically $0.2\% \sim 0.3\%$ with 95% confidence. From the fitted band gap, we theoretically calculate the corresponding lattice depth. Linear fitting of the predicted lattice depth against the lattice power PID setpoint results in a lattice depth calibration with respect to the setpoint (Supplementary Fig. 3c).

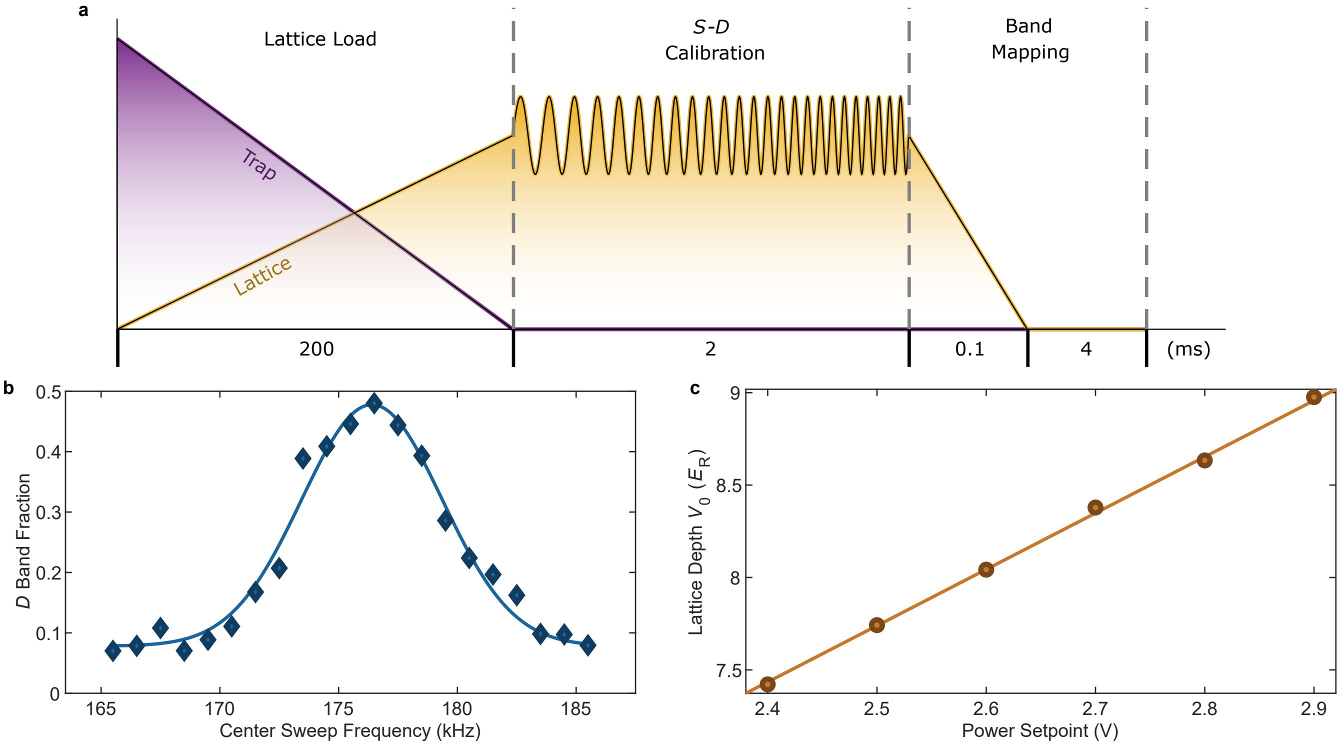

Supplementary Fig. 3. **Lattice depth calibration.** (**a**) Experimental sequence for lattice depth calibration. The dipole trap is ramped down and the lattice is ramped up over 200 ms, followed by a resonant amplitude modulation with a 1 kHz frequency sweep over 2 ms. Band-mapping separates the S and D band populations for detection. (**b**) D band population as a function of center sweep frequency (blue diamonds). A Gaussian fit (blue line) indicates the resonance, which corresponds to the gap between the S and D bands. (**c**) The experimentally measured band gap is compared to numerical predictions to extract the lattice depth V_0 as a function of lattice power setpoint (rust circles), from which we extract a linear fit (rust line) which determines the horizontal error bars in Fig. 2b in the main text.

4.2. Force

To measure the force effecting a Bloch oscillation, it suffices to measure the Bloch oscillation frequency $f_B = 1/T_B = \mathcal{F}/2\hbar k_L$. To do this, we prepare the atoms in the ground band of a deep ($12.3\,E_R$) static lattice, initiate a Bloch oscillation with the gradient coils at the chosen setpoint, and measure the quasimomentum using band-mapping after a variable hold time in the lattice. The Bragg scattering at the Brillouin zone edge combined with the nearly-constant dispersion relation of the S band in the deep lattice (tight-binding) limit gives rise to a sawtooth-like dependence

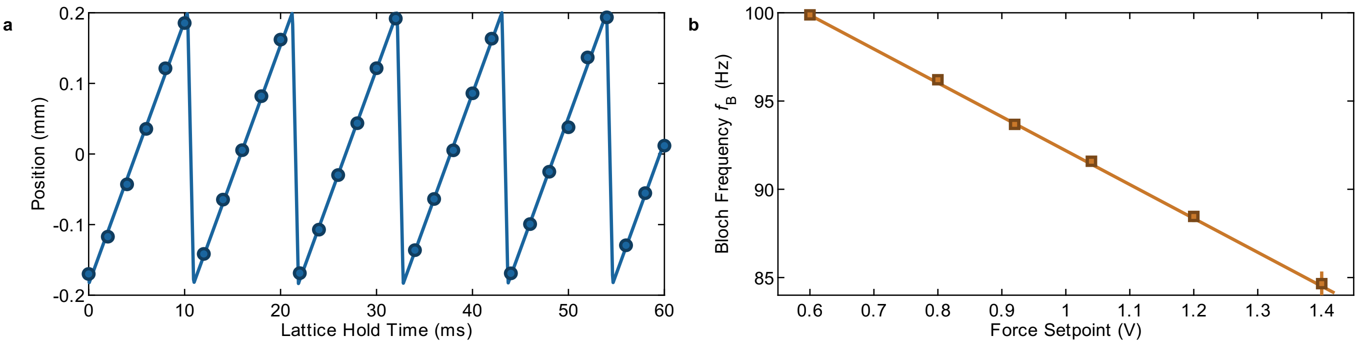

Supplementary Fig. 4. **Force Calibration.** (**a**) Fitted atomic positions (blue circles) after band-mapping during a Bloch oscillation with frequency $91.6 \pm 0.2\,\text{Hz}$ in a static lattice with variable hold time. The triangle-wave fitting curve (blue line) extracts the Bloch frequency. (**b**) Fitted Bloch oscillation frequency as a function of gradient coil setpoint (rust squares). Vertical error bars result from the aforementioned triangle-wave fits. The linear fit (rust line) determines the horizontal error bars in all the interferometric fringes with respect to Bloch period.

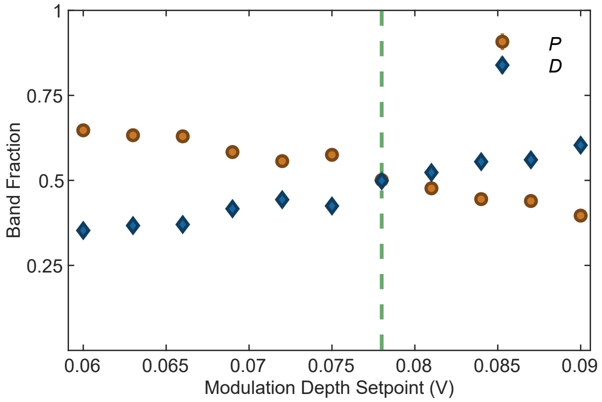

Supplementary Fig. 5. **Calibration of modulation depth**. Relative P and D band populations after the atoms undergo Landau-Zener tunneling at the first avoided crossing. By scanning the modulation depth, we identify the point at which the population split between the two bands is equal. The green dashed line indicates the calibrated modulation depth $\delta V = 0.35\,E_\text{R}$, where our Landau-Zener beamsplitter is effectively set to a 50-50 splitting. Measurements taken at a lattice depth of $V_0 = 8.45\,E_\text{R}$ and modulation frequency $127.4383\,\text{kHz}$. The modulation depth set point is scanned over a range which corresponds to $\delta V = 0.28\,E_\text{R}$ to $\delta V = 0.40\,E_\text{R}$.

of momentum on the lattice hold time (Supplementary Fig. 4a), from which we can extract the Bloch oscillation frequency. Performing this same measurement across a range of force setpoints results in a linear fit (Supplementary Fig. 4b) with which to determine the Bloch frequency and thus the real force over the range of setpoints used for the interferometry experiments.

4.3. Modulation Depth

To calibrate the beamsplitting fraction of the two Landau-Zener avoided crossings, we ramp down our P-D modulation once the atoms reach the Floquet-Brillouin zone edge at $q = \hbar k_\text{L}$ so that they undergo only the first avoided crossing. We perform the remainder of the normal interferometer experimental sequence and measure the two output port populations. We empirically select the modulation depth for which the output populations are equal, ensuring that both beamsplitters in the full interferometer sequence set the Landau-Zener transition probability $\mathcal{P} = \exp(-2\pi\delta) = 0.5$.

However, we only calibrate this modulation depth for the magic condition; since we fix δV, the beamsplitting fraction $\mathcal{P}$ changes slightly for other lattice depths as a result of changes to $|\varphi_{n,q_r}\rangle$ (see Eq. S12). This undoubtedly accounts for some of the loss in fringe contrast away from the magic condition (Fig. 2b in the main text).

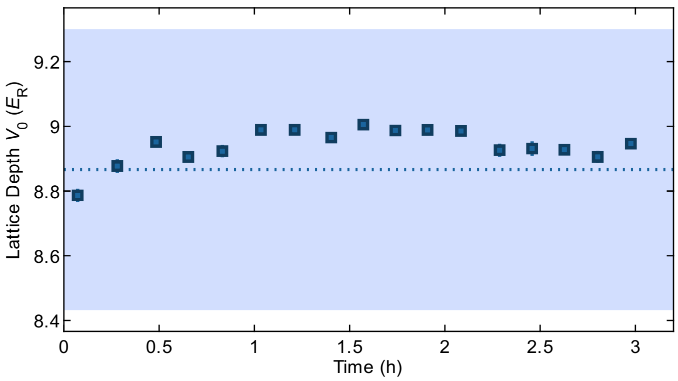

Supplementary Fig. 6. **Lattice Depth Stability.** Lattice depth as measured by the S-D modulation spectroscopy calibration procedure over three hours (blue squares), compared to the numerically predicted magic depth (blue dotted line) and its $\pm\pi/4$ interferometer phase tolerance (light blue region) for a $\Delta q = 0.4\hbar k_{\mathrm{L}}$ loop. Error bars arise from the fit of the spectroscopy resonance.

5. STABILIZATION

Key parameters of the experiment are actively stabilized during interferometer operation; here we present some details of those feedback loops.

Lattice Laser Power: Our optical lattice beam is produced by an acousto-optic modulator (AOM), so its optical power is controlled by the amplitude of the AOM's RF drive. Since our interferometric phase is sensitive to the lattice depth, we stabilize the laser power using PID feedback from a large-area photodiode. We filter the photodiode output through a 10 kHz low-pass, since the PID is active during our ~ 100 kHz amplitude modulation.

Lattice Laser Pointing: While we do not use a cavity to stabilize our optical lattice mode, we do perform active drift correction of optical lattice pointing. This is done with an active beam stabilization system from MRC Systems GmbH for the incoming lattice beam and its retroreflection; each beam's position is measured by a position-sensitive device (PSD) sensor and steered by a piezo-actuated mirror mount. Between experimental runs, the optical lattice is flashed on for 250 ms for the MRC PID to recalibrate the pointing of the input and retroreflected beams of the optical lattice, in turn. Careful alignment into the sensors during setup avoids the need to re-align the beams manually. As shown in Supplementary Fig. 6, the combination of lattice laser power and pointing stabilization gives rise to a stable lattice depth with a drift less than $0.1\,E_{\mathrm{R}}$ after the initial warm-up stage. This data set was measured via a series of S-D modulation spectroscopy scans (as demonstrated in Supplementary Fig. 3) over three hours.

Gradient Coil Current: The force responsible for Bloch oscillations is provided by a magnetic field gradient on the order of 1 G/cm produced by a coil whose current is actively stabilized. The current is measured by a transducer (Danisense DS50UB-10V) with a feedback loop implemented through a PI controller (Newport LB1005-S). The controller stabilizes the coil's current using a shunt MOSFET (IXYS IXFN140N20P) in parallel with the coil.

6. FRINGE CONTRAST REDUCTION

A current limitation of interferometer performance is that the fringe contrast decreases as the enclosed space-time area grows. Numerical simulations of a $\Delta q = \hbar k_{\mathrm{L}}$ loop that incorporate the axial Rayleigh range of the lattice laser beam and mean-field interactions show no significant change in fringe visibility, indicating that these effects are not responsible for the observed low contrast. For the largest loop ($\Delta q = \hbar k_{\mathrm{L}}$), the output imbalance fluctuates around $\mathcal{I} = 0 \pm 0.1$ instead of showing shot-to-shot noise between $\mathcal{I} = \pm 1$. Therefore, we likewise exclude noise in the lattice depth or the applied force at frequencies below the inverse loop period as a dominant mechanism.

We conclude that the most likely factor leading to reduced contrast is imperfect loop closure. A non-uniform applied force will differentially accelerate atoms in the P and D bands, so that the two arms of the interferometer no longer intersect perfectly at the second beamsplitter. The resulting spatial mismatch is negligible for small loops but increases with enclosed space-time area, naturally explaining the observed contrast decay at large Δq. Measurements of a 2 Hz residual magnetic field curvature along the lattice direction x and accompanying numerical simulations confirm that the axial field curvature has a negligible effect, leaving transverse inhomogeneities as the leading candidate. Time-of-flight measurements suggest that transverse forces are on the order of Mg along the y and z axes. Such a large magnetic force is likely non-uniform across the millimeter extent that the atoms travel, and even a small gradient

may be sufficient to drive transverse excitations leading to a differential position shift between condensates at the end of the interferometer loop, resulting in imperfect closure. If this is the case, identifying and compensating for these transverse gradients should improve contrast for larger loop areas and pave the way for further scaling of the interferometer and its performance. Likewise, transverse force fluctuation arising from unwanted lattice acceleration will by the same mechanism degrade the interferometer contrast; this in turn can be addressed with mode filtering via a resonant cavity.

7. INITIAL MOMENTUM AND PULSE DURATION TOLERANCE

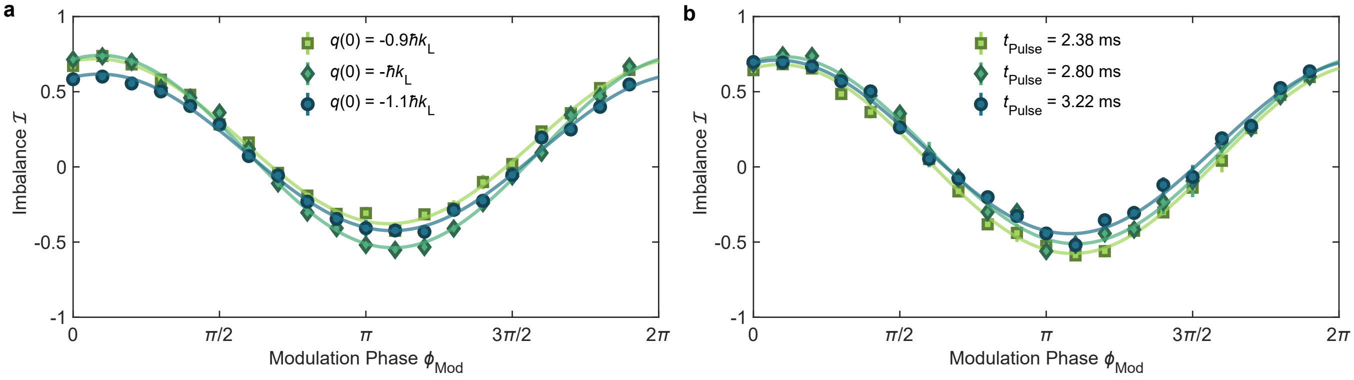

Supplementary Fig. 7. **Insensitivity to initial momentum and pulse duration**. (**a**) Output population imbalance as a function of ϕ_{Mod} for various values of matter-wave quasimomentum $q(0)$ at the beginning of S-P modulation. Apart from the varying quasimomentum, the interferometer protocol is identical to Fig. 4e in the main text. The applied force is fixed at $T_{\mathrm{B}} = 10.70\,\mathrm{ms}$ and the interferometer loop size is $0.4\hbar k_{\mathrm{L}}$. (**b**) Output population imbalance as a function of ϕ_{Mod} for various beamsplitter pulse durations. The applied force and loop size are the same as in (a). As in Fig. 4e in the main text, the two modulation pulses are applied symmetrically across the Brillouin zone edge.

Our Floquet-Bloch matter-wave interferometer employs Landau-Zener beamsplitters that provide intrinsic tolerance against fluctuations in both initial quasimomenta $q(0)$ and pulse durations t_{pulse}. As long as the remaining duration of the modulation pulse exceeds the Landau-Zener transition timescale when the matter-wave reaches the resonant quasimomentum q_{r}, the matter-wave splitting remains faithfully 50-50. Figure. 7a shows the measured interference fringes with $\pm 0.1\hbar k_{\mathrm{L}}$ variations of the initial quasimomenta at the beginning of the S-P modulation. The resulting fringes exhibits only a 0.06 contrast variation, while the fitted interferometer phases remain nearly unchanged. In Supplementary Fig. 7b, we instead vary the modulation pulse durations by 15% and observe a modest 0.025 change of the contrast.

8. EXTENDING THE LOOP SIZE

Here we present numerical results demonstrating that the force response continues to scale up when the interferometric loop includes multiple Bloch oscillations. For simplicity, we adopt the pulsed P-D interferometry scheme, with separation and recombination pulses applied when the quasimomentum reaches $\hbar k_{\mathrm{L}} - \Delta q/2$ and $\hbar k_{\mathrm{L}} + \Delta q/2$. In large-loop configurations, magic band structures can require smaller lattice depths, leading to increased Landau-Zener tunneling from the D to F band at the Brillouin zone edge. To mitigate this unwanted transition, we assume a reduced force of $\mathcal{F} \approx 0.2\,Mg$ while adjusting the P-D modulation strength to maintain 50-50 splitting. Figure 8a shows a simulated interference fringe for $\Delta q = 5.4\hbar k_{\mathrm{L}}$ and $V_0 = 5.63\,E_{\mathrm{R}}$ yielding a magic condition. The top axis indicates a much larger force response compared to the smaller loops in Fig. 3 in the main text. Although large-loop interference has not yet been experimentally observed due to contrast limitations (see section 6), the measured matter-wave trajectory reveals an enlarged spacetime area as the atoms undergo multiple Bloch oscillations (Supplementary Fig. 8b).

When Δq approaches an integer multiple of $2\hbar k_{\mathrm{L}}$, the resonant quasimomenta lie near the Brillouin zone edge or center where the P-D coupling vanishes. A magic band structure is absent in such conditions, resulting in the gaps in Supplementary Fig. 8c and 8d. As the loop size increases, the magic depth V_0^{M} oscillates and eventually converges to a constant value (Supplementary Fig. 8c), which corresponds to the stationary point of the differential dynamical phase integrated over the full Brillouin zone. Figure 8d shows that the force response also oscillates with loop size.

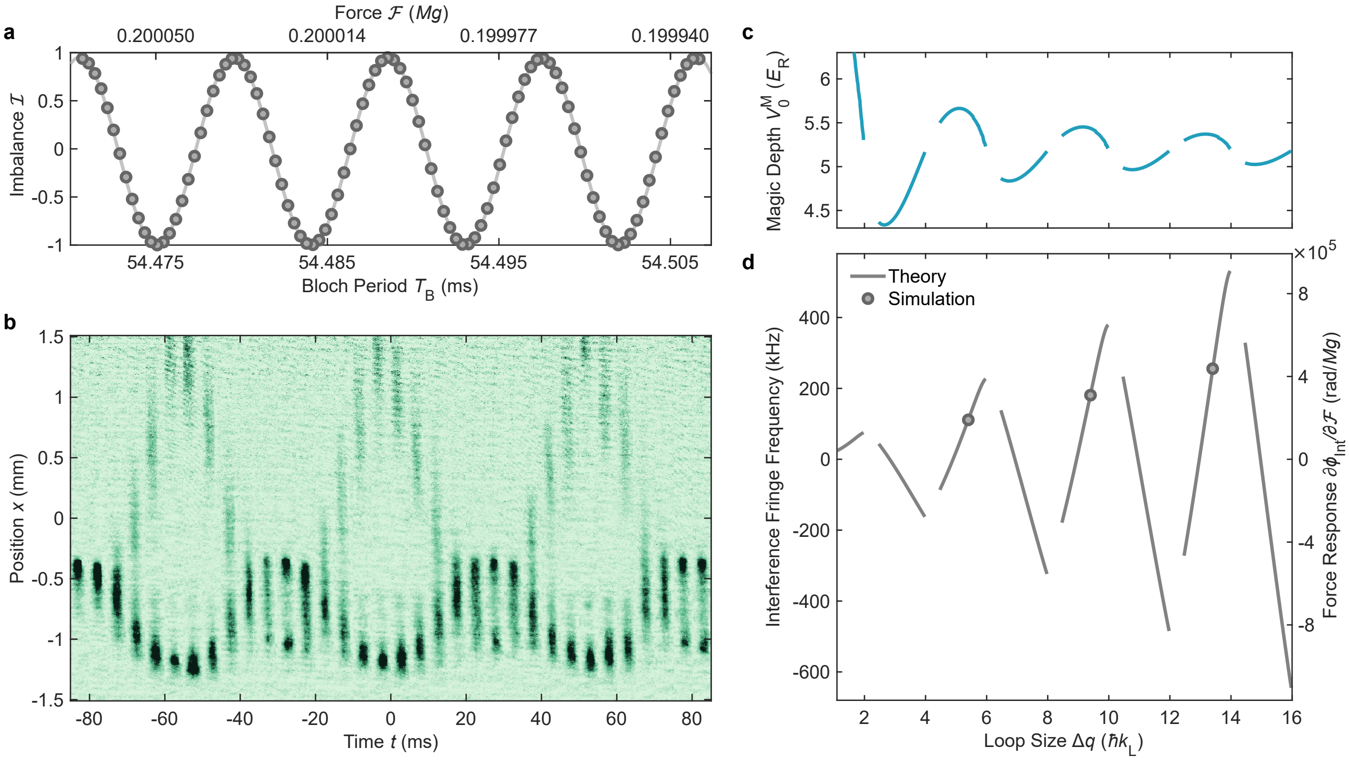

Supplementary Fig. 8. **Extending the loop size in the weak-force regime**. (**a**) Numerically simulated interference fringe for loop size $\Delta q = 5.4\,\hbar k_{\mathrm{L}}$ when $\mathcal{F} \approx 0.2\,Mg$. The solid line is a sinusoidal fit. The lattice depth $V_0 = 5.63\,E_{\mathrm{R}}$ yields a magic band structure. As in Fig. 4a in the main text, two modulation pulses with $t_{\text{pulse}} = 12.5\,\text{ms}$ are applied symmetrically across the Brillouin zone edge when $q = -1.7\,\hbar k_{\mathrm{L}},\ 3.7\,\hbar k_{\mathrm{L}}$. (**b**) Experimental demonstration of the atoms traversing a $\Delta q = 5.4\,\hbar k_{\mathrm{L}}$ P-D interferometer loop when $\mathcal{F} \approx 0.2\,Mg$. The lattice depth is kept at $9\,E_{\mathrm{R}}$ to prevent D band atoms from moving out of the field of view. (**c**) Theoretically calculated magic depth as the loop size extends to include multiple Bloch oscillations, assuming the interferometer loop is symmetric about $q = \hbar k_{\mathrm{L}}$. The interband coupling due to amplitude modulation vanishes at the Brillouin zone edge and center, so the existence of an upper bound on feasible modulation amplitudes results in the disappearance of a magic structure for a range of loop sizes near integer multiples of $2\hbar k_{\mathrm{L}}$ (see section 2). (**d**) Interferometric response as a function of loop size as defined by the fringe frequency. The sign of the fringe frequency represents the sign of the interferometer phase. Data points represent fit results from numerically simulated fringes, and the solid line is the theoretical prediction obtained from the fit-parameter-free analytical theory. The corresponding force response is calculated at $\mathcal{F} = 0.2\,Mg$.

At certain loop sizes, destructive interference between the P and D band dynamical phases leads to vanishing force response, which could potentially be combined with echo techniques for AC force sensing. Despite the oscillations, the overall force response trends up, indicating the expected enhanced sensitivity for longer trapping durations.

9. ACCELERATION SENSITIVITY

Fundamentally, the sensitivity we can achieve is limited by the standard quantum limit (SQL) for uncorrelated atoms, which gives a phase estimation error bound of $\delta\phi_{\text{Int}}^{\text{SQL}} = 1/\mathcal{C}\sqrt{N}$, where N is the number of atoms and $\mathcal{C}$ is the measured contrast. The single-shot SQL of the acceleration sensitivity is then given by

$$\frac{\delta a^{\text{SQL}}}{a} = \frac{\delta\phi_{\text{Int}}^{\text{SQL}}}{\phi_{\text{Int}}} = \frac{1}{\mathcal{C}\sqrt{N}}\frac{\mathcal{F}}{\hbar\mathcal{A}}, \tag{S48}$$

where

$$\mathcal{A}(V_0,\omega) = \int_{k_{\mathrm{r}}(V_0,\omega)}^{k'_{\mathrm{r}}(V_0,\omega)=k_{\mathrm{r}}+\Delta k} \mathrm{d}k\,\left[\tilde{\Omega}_{\mathrm{U},k}(V_0) - \tilde{\Omega}_{\mathrm{L},k}(V_0)\right] \tag{S49}$$

denotes the integrated band energy difference controlled by the lattice depth V_0 and the modulation frequency ω; the quasimomentum and quasienergy are normalized by $\hbar$:

$$k_{\mathrm{r}} = \frac{q_{\mathrm{r}}}{\hbar}, \qquad \Delta k = \frac{\Delta q}{\hbar}, \qquad \tilde{\Omega}_{\mathrm{U},k} = \frac{\tilde{E}_{\mathrm{U},\hbar k}}{\hbar}, \qquad \tilde{\Omega}_{\mathrm{L},k} = \frac{\tilde{E}_{\mathrm{L},\hbar k}}{\hbar}. \tag{S50}$$

If we assume the beamsplitters couple the two bands near the Brillouin zone's edge or center, Eq. (S48) can be approximated as

$$\frac{\delta a^{\mathrm{SQL}}}{a} \approx \frac{1}{\mathcal{C}T\sqrt{N}\left(\bar{\Omega}_{\mathrm{U}} - \bar{\Omega}_{\mathrm{L}}\right)}, \tag{S51}$$

where T is the interrogation time and $\bar{\Omega}_{\mathrm{U(L)}}$ represents the average band energy of the upper (lower) band divided by $\hbar$. When $N = 10^5$, $\mathcal{F} = 0.2\,Mg$, and the loop size $\Delta q = 15.8\hbar k_{\mathrm{L}}$, the above SQL gives a 1.7×10^{-8} relative acceleration sensitivity with a single shot, assuming a perfect contrast. The corresponding interrogation time is 431 ms.

We can estimate the acceleration sensitivity from the phase measurement in Fig. 4f in the main text. The fit gives a statistical error of 0.019 rad (68% confidence interval) for the interferometer phase, after a 20 min phase measurement. This corresponds to a 1.01×10^{-4} relative acceleration sensitivity under the experimental parameters $T_{\mathrm{B}} = 10.56$ ms and $\Delta q = 0.4\hbar k_{\mathrm{L}}$. To enhance the sensitivity, we will have to enlarge Δq, which requires a proper handling of atomic transverse motions.

10. SOURCES OF SYSTEMATIC ERROR AND NOISE

In this section, we discuss possible sources of systematic error and noise. It will be our future work to reduce the systematic errors and noise for demonstrating precision measurements.

10.1. Lattice Depth

To evaluate the effects of lattice depth uncertainty, we rewrite the interferometer phase as (ignoring the Stokes phase and assuming perfect beamsplitting):

$$\phi_{\mathrm{Int}} = \frac{1}{\hbar\mathcal{F}} \int_{q_{\mathrm{r}}}^{q_{\mathrm{r}}' = q_{\mathrm{r}} + \Delta q} \mathrm{d}q \left(\tilde{E}_{\mathrm{U},q} - \tilde{E}_{\mathrm{L},q}\right) = \frac{\hbar}{\mathcal{F}} \mathcal{A}(V_0, \omega), \tag{S52}$$

where $\mathcal{A}(V_0, \omega)$ is defined in Eq. (S49). Suppose we determine the absolute phase ϕ_{Int} from interference fringes with an uncertainty $\sigma_{\phi_{\mathrm{Int}}}$, the acceleration a can then be determined as

$$a = \frac{\mathcal{F}}{M} = \frac{\hbar}{M} \frac{\mathcal{A}(V_0, \omega)}{\phi_{\mathrm{Int}}}, \tag{S53}$$

with the uncertainty

$$\frac{\sigma_a}{a} = \sqrt{\left(\frac{\sigma_{\phi_{\mathrm{Int}}}}{\phi_{\mathrm{Int}}}\right)^2 + \left(\frac{\sigma_{\mathcal{A}}}{\mathcal{A}}\right)^2 + \left(\frac{\sigma_{\hbar/M}}{\hbar/M}\right)^2}. \tag{S54}$$

Since the ratio $\hbar/M$ can be measured to a precision far beyond other sources of errors, we will ignore the contribution of $\sigma_{\hbar/M}$. The factor $\mathcal{A}(V_0, \omega)$ is proportional to the lattice wave vector k_{L} and the recoil frequency $E_{\mathrm{R}}/\hbar$, both of which can be determined with a high precision. In addition, it also depends on the control parameters ω and V_0. ω is an RF frequency which can be determined very precisely. While V_0 is in general less precisely known, the contribution due to uncertainty in V_0 is reduced by the magic band structure. Near the magic condition where $\partial\mathcal{A}/\partial V_0\,|_{V_0 = V_0^{\mathrm{M}}} = 0$, the uncertainty σ_{V_0} only has a second-order contribution to $\sigma_{\mathcal{A}}$. Therefore, we can rewrite Eq. (S54) as

$$\frac{\sigma_a}{a} \approx \sqrt{\left(\frac{\sigma_{\phi_{\mathrm{Int}}}}{\phi_{\mathrm{Int}}}\right)^2 + \left(\frac{\sigma_{V_0}}{V_0^{\mathrm{T}}}\right)^4}, \tag{S55}$$

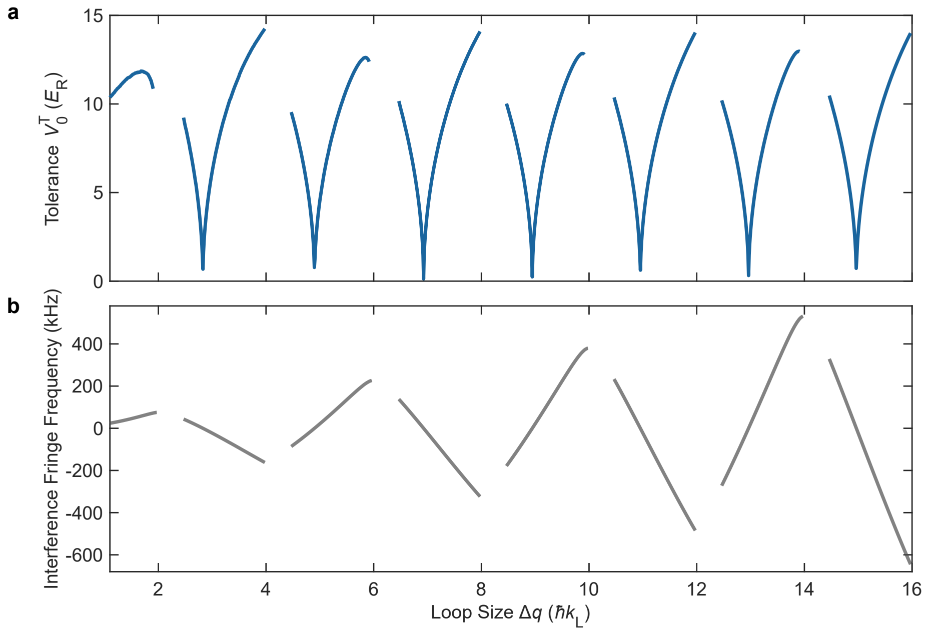

Supplementary Fig. 9. **Insensitivity to lattice depth uncertainty.** (**a**) Lattice depth uncertainty tolerance V_0^{T} as a function of loop size Δq. (**b**) Interference fringe frequency versus loop size Δq.

where

$$V_0^{\mathrm{T}} = \sqrt{2\left|\frac{\mathcal{A}}{\partial^2\mathcal{A}/\partial V_0^2|_{V_0=V_0^{\mathrm{M}}}}\right|} \tag{S56}$$

characterizes the tolerance against lattice depth uncertainty. Eq. (S55) tells us the requirement for lattice depth uncertainty is not stringent if one wants to demonstrate precision measurements. The underlying idea is similar to the magic wavelength approach in optical lattice clocks, where the first-order differential light-shift from the lattice is canceled, and the effects of lattice intensity fluctuation are minimized.

To provide a sense of how precisely we need to determine V_0, we plot V_0^{T} as a function of loop size in Supplementary Fig. 9. We notice that V_0^{T} is typically about $10\,E_{\mathrm{R}}$ for loop sizes at which the force response is peaked. This implies that if we want to reach a relative measurement accuracy of 10^{-6}, we only need to reduce σ_{V_0} below $0.01E_{\mathrm{R}}$, which is experimentally accessible.

In practice, the uncertainty σ_{V_0} is dependent on lattice depth calibration as well as lattice fluctuations. As presented in section 4.1, the approach we took for lattice depth calibration is amplitude-modulation spectroscopy, which typically gives better precision than Kapitza-Dirac diffraction. The statistical uncertainty of the calibrated lattice depth at a given PID control voltage is about $0.2\% \sim 0.3\%$ with 95% confidence. The major systematic error for this calibration is from atom number counting and initial quasimomentum uncertainty. We believe the spectroscopic resolution of this approach can be further enhanced by reducing the detection noise and cooling the atomic cloud even more. On the other hand, lattice fluctuations contribute more significantly to σ_{V_0}. As shown in Supplementary Fig. 6, the typical lattice depth drift is currntly about $0.1\,E_{\mathrm{R}}$ shot-to-shot in our experiments. We believe this drift is mostly induced by laser pointing noise. With a more compact setup using fiber collimated lattice beams, the fluctuations can be greatly reduced.

10.2. Modulation Depth

The modulation depth δV contributes to the interferometer fringe in two ways. First, it determines the (ideal) fringe contrast. A fluctuating modulation depth would modestly reduce the fringe contrast. Recall that the ideal contrast C depends on the LZ transition probability $\mathcal{P}$ as $C = 4\mathcal{P}(1-\mathcal{P})$. Since $\partial C/\partial\mathcal{P}|_{\mathcal{P}=0.5} = 0$, the uncertainty in $\mathcal{P}$ (so as the uncertainty in δV) only has a second-order contribution to the uncertainty in C. As an example, a 10% peak-to-peak fluctuation in δV only leads to a 0.5% reduction in the contrast. Secondly, the change in the modulation

depth leads to a shift in the Stokes phase. Because the Stokes phase is bounded as $\phi_{\rm Sto} \in [-\pi/2, -\pi/4]$, the phase shift induced by a fluctuating δV is usually small. For example, a 10% peak-to-peak fluctuation in δV would result in a 0.02 rad shift in the Stokes phase. To achieve extreme high precision, users of this technique should carefully reduce the fluctuations in δV using, for example, an extra layer of laser power PID that stabilizes the laser power into the modulated AOM.

10.3. Acceleration Gradient

Next, we consider the effects of a spatially inhomogeneous force (acceleration). Intuitively, a spatially varying force can both induce a phase shift and reduce the contrast. To quantify these effects, we assume the total force can be written as $\mathcal{F} + M\gamma x$. In addition, we assume γ is small such that the Bloch Theorem is approximately valid and the dispersion relation $\tilde{E}_{n,q}$ still holds. The center-of-mass motions of the two separated clouds are governed by the following classical equations:

$$\frac{\mathrm{d}q}{\mathrm{d}t} = \mathcal{F} + M\gamma x, \tag{S57}$$

$$\frac{\mathrm{d}x}{\mathrm{d}t} = \frac{\mathrm{d}\tilde{E}}{\mathrm{d}q}, \tag{S58}$$

where $x(t)$ and $\tilde{E}(q)$ represent the center-of-mass trajectory and the Floquet-Bloch band energy of the upper or lower band atomic cloud, respectively. These are nonlinear equations, and we will attempt to solve them perturbatively. Ignoring γ, the unperturbed solution is

$$q^{(0)}(t) = q_{\rm r} + \mathcal{F}t, \tag{S59}$$

$$x^{(0)}(t) = x_{\rm r} + \frac{\tilde{E}(q^{(0)}(t))}{\mathcal{F}}, \tag{S60}$$

where $q_{\rm r}$, $x_{\rm r}$ denote the initial quasimomentum and position, respectively. We assume $q(t)$ and $x(t)$ can be written as a perturbation series of γ,

$$q(t) = q^{(0)}(t) + M\gamma q^{(1)}(t) + O(\gamma^2), \tag{S61}$$

$$x(t) = x^{(0)}(t) + M\gamma x^{(1)}(t) + O(\gamma^2). \tag{S62}$$

Plugging the above ansatz into Eqs. (S57) and (S58), we find

$$\frac{\mathrm{d}q^{(1)}}{\mathrm{d}t} = x^{(0)}(t), \tag{S63}$$

$$\frac{\mathrm{d}x^{(1)}}{\mathrm{d}t} = q^{(1)}(t) \times \left.\frac{\mathrm{d}^2\tilde{E}}{\mathrm{d}q^2}\right|_{q=q^{(0)}(t)}. \tag{S64}$$

Solving the above equations gives

$$q^{(1)}(t) = x_{\rm r}t + \frac{1}{\mathcal{F}^2}\int_{q_{\rm r}}^{q_{\rm r}+\mathcal{F}t} \mathrm{d}q\, \tilde{E}(q), \tag{S65}$$

$$x^{(1)}(t) = \int_0^t \mathrm{d}t'\, q^{(1)}(t') \times \left.\frac{\mathrm{d}^2\tilde{E}}{\mathrm{d}q^2}\right|_{q=q_{\rm r}+\mathcal{F}t'}. \tag{S66}$$

Eq. (S65) shows that the quasimomentum shift induced by the acceleration gradient γ is dependent on the dispersion relation $\tilde{E}(q)$, which implies that the two interferometer arms have different final quasimomenta. This differential quasimomentum shift leads to a contrast reduction that can be evaluated from the final quasimomentum overlap of the two atomic clouds. In the following, we consider an interferometry setup as mentioned before: $N = 10^5$, $\mathcal{F} = 0.2\,Mg$, and $\Delta q = 15.8\hbar k_{\rm L}$. We also assume the final quasimomentum distributions are Gaussian functions with a $0.13\,\hbar k_{\rm L}$ full-width-half-maximum, same as the initial state. As shown in Supplementary Fig. 10(a), the fringe contrast in the presence of γ is calculated as the overlap of two Gaussian distributions displaced by the differential quasimomentum shift. We notice that γ needs to be as large as $5\,\mathrm{s}^{-2}$ to reduce the contrast by a half. Because it is

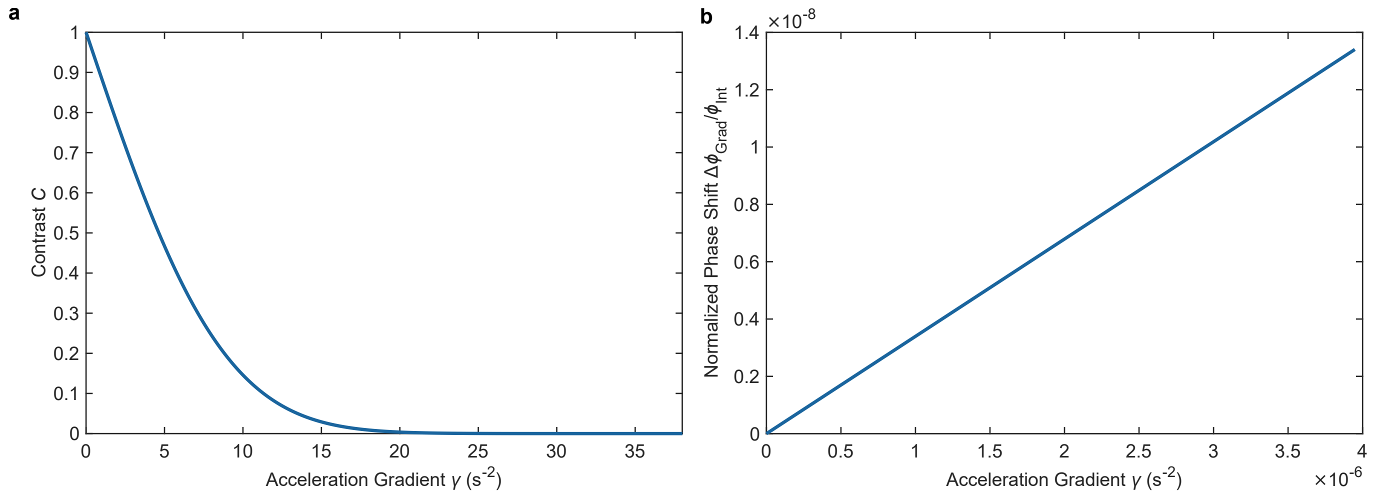

Supplementary Fig. 10. **Acceleration gradient induced effects.** (**a**) Fringe contrast as a function of γ, evaluated as the overlap of final quasimomentum distributions. Assuming $\mathcal{F} = 0.2\,Mg$ and $\Delta q = 15.8\hbar k_{\mathrm{L}}$. (**b**) Acceleration gradient induced phase shift, estimated from the change of the spacetime area under the same interferometry parameters.

experimentally straightforward to control γ below $1\,\mathrm{s}^{-2}$ (at which the contrast is only reduced to 0.88), we believe the gradient-induced contrast reduction is less relevant to a consideration of force sensitivity.

Since the interferometer phase scales with the enclosed spacetime area, we estimate the gradient-induced phase shift $\Delta\phi_{\mathrm{Grad}}$ from the change of the spacetime area given by integrating Eq. (S66). For simplicity, we assume $x_{\mathrm{r}} = 0$, which implies the force reference position is where the atomic cloud is split. Supplementary Fig. 10(b) shows that when $\gamma = 3 \times 10^{-6}\,\mathrm{s}^{-2}$ (about the Earth's gravity gradient), the induced relative phase shift is about 10^{-8}, which is comparable to the SQL of the phase sensitivity. This phase shift is a systematic error that can be treated if γ is measured independently. Indeed, in relevant work of freefall atom interferometry (Peters *et al.*, 2001 Metrologia 38 25), the authors obtained an absolute measurement of gravity after the correction from the gravity gradient measured by LaCoste-Romberg spring-type gravimeters.

10.4. Interaction

Based on our force calibration, the typical magnetic field gradient along the lattice axis is $1.25\,\mathrm{G/cm}$. At the zero-crossing ($543.6\,\mathrm{G}$), the slope of the scattering length versus the magnetic field is $0.071\,a_0/\mathrm{G}$, where a_0 is the Bohr radius. In our experiments, the atomic position deviation from where we calibrated the zero-crossing is about $0.5\,\mathrm{mm}$, which maximally leads to a scattering length of $a_{\mathrm{s}} \pm 0.004\,a_0$ induced by the field gradient. Considering that the typical atomic density is $n_{\mathrm{atom}} = 1.4 \times 10^{18}\,\mathrm{m}^{-3}$, the induced mean-field energy shift will be $4\pi\hbar^2 a_{\mathrm{s}} n_{\mathrm{atom}}/M \approx 5\,\mathrm{mHz}$ per particle. This is a very small shift on the time scales of our experiment.

The Feshbach magnetic field is calibrated using atomic microwave spectroscopy with a precision of about $100\,\mathrm{mG}$, which corresponds to a residual scattering length of $0.007\,a_0$. This residual interaction can cause a $9\,\mathrm{mHz}$ mean-field energy shift which is negligible in our experiments.

10.5. Other sources

Detection noise. Since we use absorption imaging to determine the atom number, the background photon shot noise could dominate quantum projection noise.

Field gradient noise. The force that our interferometer is sensitive to is produced by magnetic coils. Although the current flowing into the coils is PID controlled, the residual noise of 2×10^{-4} can potentially limit how far we can push the sensitivity.

Transverse motion. As discussed in section 6, we believe the transverse motions of the atoms are the major source of contrast reduction. These motions can be induced by the transverse magnetic field curvature or the transverse jitter of the lattice beam.

11. USING HEAVIER ATOMS

In addition to the straightforward change in the amplitude of Bloch oscillations, the most significant consequence of using heavier atoms will be the increased possibility of LZ tunneling to other bands. In our scheme, a key limiting process will be LZ tunneling from the D band to the F band at the Brillouin zone edge; such tunneling represents a "leak" which removes atoms from the interferometer loop. Here we discuss how this process depends on atomic mass.

We assume the lattice laser wavelength does not change when using different species. With a larger atomic mass, the recoil energy E_{R} becomes smaller, rescaling the static band structure. Therefore, the proportionality $V_0^{\mathrm{M}}/E_{\mathrm{R}}$ remains constant for a given loop size. We can define the normalized magic depth as $\tilde{V}_0^{\mathrm{M}} = V_0^{\mathrm{M}}/E_{\mathrm{R}}$. In the following, we assume the interferometer operates at the fixed $\tilde{V}_0^{\mathrm{M}}$ for a given loop size when we consider different atomic species. The LZ theory tells us that the transition probability through a Bloch band gap is given by

$$\mathcal{P}_{\mathrm{Bloch}} = \mathrm{e}^{-1/\kappa},\ \kappa = \frac{8}{\pi^2}\frac{\hbar\omega_{\mathrm{B}}/E_{\mathrm{R}}}{(\Delta/E_{\mathrm{R}})^2}, \tag{S67}$$

where Δ is the band gap between D and F and $\omega_{\mathrm{B}} = 2\pi/T_{\mathrm{B}}$ is the Bloch frequency. For ^{7}Li, the above equation gives $\mathcal{P}_{\mathrm{Bloch}} = 6.2 \times 10^{-11}$ through the D-F gap at the Brillouin zone edge, when $V_0 = 8.45\,E_{\mathrm{R}}$ and $T_{\mathrm{B}} = 10.7\,\mathrm{ms}$. Notice that the band gap scales with E_{R}, so that Δ/E_{R} remains constant. Therefore, $\kappa \propto M^2 a$ where a is the acceleration. This tells us that when we operate the interferometer under the magic condition, the range of acceleration we can measure scales with $1/M^2$ if we want to maintain a low coupling rate to unwanted bands. Fortunately, this problem is not in principle unsolvable. In a manner similar to the measurements shown in Fig 4c and d, we can superimpose another modulation frequency that couples the D band to the S band near the Brillouin zone edge, such that D band atoms are shelved in S when they are close to $q = k_{\mathrm{L}}$, avoiding the tunneling leakage and allowing the use of heavier atoms. This scheme will of course modify the interferometer phase and require a somewhat different magic condition.

Because the recoil energy E_{R} is smaller for heavier atoms, the amount of optical power required under the magic condition is smaller if we assume a fixed laser detuning and atomic dipole matrix element. Meanwhile, the SQL of the acceleration sensitivity given by Eq. (S51) scales with M for a fixed interrogation time T. Equivalently, it takes a longer interrogation time for heavier atoms to reach the same sensitivity as lighter atoms.

SUPPLEMENTARY REFERENCES

[1] T. N. Ikeda, S. Tanaka, and Y. Kayanuma, *Floquet-Landau-Zener interferometry: Usefulness of the Floquet theory in pulse-laser-driven systems*, Physical Review Research **4**, 033075 (2022).

[2] D. W. Hone, R. Ketzmerick, and W. Kohn, *Time-dependent Floquet theory and absence of an adiabatic limit*, Physical Review A **56**, 4045 (1997).

[3] M. Holthaus, *Floquet engineering with quasienergy bands of periodically driven optical lattices*, Journal of Physics B: Atomic, Molecular and Optical Physics **49**, 013001 (2016).

[4] L. Landau, *Zur theorie der energieübertragung. I*, Physikalische Zeitschrift der Sowjetunion **1**, 88 (1932).

[5] L. Landau, *Zur theorie der energieübertragung. II*, Physikalische Zeitschrift der Sowjetunion **2**, 46 (1932).

[6] C. Zener, *Non-adiabatic crossing of energy levels*, Proceedings of the Royal Society of London. Series A, Containing Papers of a Mathematical and Physical Character **137**, 696 (1932).

[7] S. Shevchenko, S. Ashhab, and F. Nori, *Landau–Zener–Stückelberg interferometry*, Physics Reports **492**, 1 (2010).

[8] O. V. Ivakhnenko, S. N. Shevchenko, and F. Nori, *Nonadiabatic Landau–Zener–Stückelberg–Majorana Transitions, Dynamics, and Interference*, Physics Reports **995**, 1 (2023).

[9] J. Zak, *Berry's phase for energy bands in solids*, Physical Review Letters **62**, 2747 (1989).

[10] H. Breuer and M. Holthaus, *Quantum Phases and Landau-Zener Transitions in Oscillating Fields*, Physics Letters A **140**, 507 (1989).
